# ZOMES: the intriguing interplay of PCI complexes and the ubiquitin in protein homeostasis

**DOI:** 10.1038/cddis.2017.413

**Published:** 2017-08-24

**Authors:** Arno F Alpi, Aude Echalier

**Affiliations:** 1 Department of Molecular Machines and Signaling, Max-Planck-Institute for Biochemistry, Martinsried, Germany; 2 Department of Molecular and Cell Biology, University of Leicester, Leicester LE1 7RH, UK; 3 Nuffield Department of Medicine, Structural Genomics Consortium, University of Oxford, Oxford OX3 7DQ, UK

The ZOMES IX conference ‘PCI complexes and ubiquitin defining a hub for protein homeostasis’ was organised by Giovanna Serino, Rodolfo Negri, Cristina Mazzoni (Sapienza University, Italy) and Simona Polo (IFOM, Italy) and held between 14^th^ and 17^th^ February 2017 in Rome (Italy).

The aim of ZOMES, a biennial meeting since 2000, is to bring together scientists with an interest in three highly conserved families of macromolecular protein complexes collectively referred to as ‘ZOMES’: the 26S proteasome lid (LID), the COP9 signalosome (CSN), and the eukaryotic translation initiation factor 3 (eIF3). The uniqueness of this conference is that it brings together scientists working on the biology of unicellular organisms, animals and plants, at the whole organism, cellular and atomic levels, allowing to cross-fertilise research efforts, build a comprehensive picture of the field, and lay the bases for collaborative work.

Canonical ZOMES share a common architecture and exist in all multicellular organisms and most unicellulars. In the past few years, details of this architecture and important regulatory insights have been unravelled by significant molecular efforts and complemented functional investigations, deepening our understanding of the ZOMES. The ZOMES are involved in virtually all pathways and processes, making their study both fascinating and challenging. Although both the CSN and the LID directly regulate protein degradation, eIF3 contributes to protein homeostasis at the translational level. There are still major outstanding questions related to the biology of the ZOMES, but recent discoveries hold strong promises to push the boundaries of our collective understanding. ZOMES IX explored the crosstalk between ZOMES and other protein-degradation machineries.

## The proteasome, where ubiquitin defines protein fate for degradation

The 26S proteasome is the heaviest weight among the ZOMES. Recent advances in cryo-electron microscopy (EM) have revealed high-resolution structures of the human and yeast proteasomes, with a central 20S core particle, the proteolytic chamber, and one or two 19S regulatory particle(s) made of two subassemblies, the ‘base’ and LID. Substrate recruitment by the regulatory particle, with ubiquitin-binding and deubiquitylase (DUB) activities, is followed by substrate engagement and proteasome conformational switch to initiate substrate deubiquitylation and unfolding before entering the 20S core. A long-standing question in the field is the proteasome substrate specificity, especially with regard to ubiquitin chain length, linkage type, and susceptibility to DUBs. Several recent studies challenged the previous paradigm that K48-linked tetra-ubiquitin is the predominant signal for proteasome degradation. The impact of trimming by DUBs was addressed by Michael Glickman (Israel), providing evidence that cleavage of the proximal isopeptide bond is not essential for degradation by the proteasome. Moreover, the proteasome ubiquitin receptors contribute to substrate specificity, as discussed by Gali Prag (Israel), with his work on the ubiquitin receptor Rpn10.

Proteasomes, workhorses in protein degradation, are also prone to damages. Richard Vierstra (USA) focused on the importance of proteophagy to maintain a balanced proteolytic capacity. Highlighting another link with autophagy, Francesco Cecconi (Italy) described how levels of the autophagic kinase ULK1 are regulated by NEDD4L ubiquitylation and proteasomal degradation to control autophagy.

## Functional dissection of the CSN: from mechanism to biology

CSN shares striking similarities with the LID. CSN specifically deconjugates the ubiquitin-like NEDD8 from cullins. Recent structural analysis revealed that CSN autoinhibition is lifted by NEDDylated CRL binding. Nicolas Thomä (Switzerland) proposed an induced-fit activatory mechanism of the CSN by NEDDylated cullin underlying this process. Presenting further level of CSN/cullin interaction regulation, Feng Rao (China) described an inositol polyphosphate as an inter-molecular bridge mediating CSN binding to cullin complexes via CSN2. Aude Echalier (UK), however, focused her studies on CSN catalytic subunit CSN5 and its dependency on CSN6 for isopeptidase activity. Surely, the comprehensive analysis of CSN significantly aided the development of a selective CSN5 inhibitor with anti-proliferative activities, presented by Martin Renatus (Switzerland).

Studies of individual CSN components revealed CSN functional and regulatory roles. Michal Sharon (Israel) gave a thought-provocative talk presenting the functional characterisation of the ninth CSN subunit CSNAP. The differential requirement of CSN7 variants and the CUL3 ubiquitin ligase in adipogenic differentiation was described by Wolfgang Dubiel (China) and Dawadschargal Dubiel (Germany). Tomohiko Tsuge (Japan) outlined a mechanism underlying an intrinsic function of CSN1 in RNA processing during *A. thaliana* pollen development. Further evidence of a functional role of CSN in gene regulation was provided by Daniel Chamovitz (Israel) from comprehensive transcriptomics data of *csn* mutants, highlighting a CSN global genome wide effect on *A. thaliana* epigenetic landscape.

## elF3: assembly, structure and link to metabolism

elF3, the most intricate eukaryotic initiation factor, comprising 8-subunit ZOME core complemented by eIF3-specific subunits is thought to assist in the attachment of the mRNA to the small ribosomal 40S subunit. Substantial progress was recently made regarding its molecular structure, as highlighted by Yaser Hashem (France). He reported a cryo-EM elF3 structure (ZOME core and two elF3-specific subunits), contributing to the understanding of eIF3 function. A comprehensive analysis of the human elF3 complex assembly in cells by Leos Valasek (Czech Republic) highlighted how imbalanced expression of elF3 subunits, frequently observed in cancer and developmental disorders, impacts on the overall functionality of elF3. An intriguing mechanism based on transcriptomic, proteomic, and metabolomic profiling of elF3 deletion mutants presented by Dieter A Wolf (USA, China), suggests a relationship between mRNA-specific translational control and altered energy metabolism in cancer.

## ZOMES interplay with E3 ubiquitin ligases

The CRL family controls ~20% of cellular protein homeostasis. The CRL complexity lies within their modular architecture based on one of the seven cullin scaffolding subunits and hundreds of different substrate receptor complexes. Their functional state is conformationally controlled by cullin NEDDylation. Recent discoveries suggest that NEDD8 plays additional roles in cullin function. Arno Alpi (Germany) showed that NEDDylated cullins recruit and activate Ariadne RBR E3 ligases. Furthermore, he revisited mechanisms of ubiquitylation proposing that cullin-RING E3 and Ariadne RBR E3 activities cooperate in client substrate poly-ubiquitylation.

CRL inactivation is facilitated by CSN-dependent deNEDDylation, defining CSN as a critical CRL regulator. Ning Wei (USA) presented her work describing CRL-regulated hypocotyl elongation of seedlings in response to light signals and the molecular targets of the CSN in seed germination and dormancy. Further examples of the involvement of E3 ubiquitin ligases in regulating protein levels of hormone signalling factors in planta were discussed, including a novel functional link between the COP1 ubiquitin ligase and auxin response in light-regulated development of flower organs by Davide Marzi (Italy).

## ZOMES and the expanding functions of UBLs

NEDD8 is involved in multiple pathways, including regulating CRL functions. Studies in yeast by Elah Pick (Israel) revealed a functional interplay of redox homeostasis and the NEDDylation cycle of CRLs following metabolic switches, supporting the growing evidence that the pool of ‘free’ NEDD8 and its availability for substrate conjugation is tightly regulated and linked to cellular stress responses. Claus Schwechheimer (Germany) discussed how *A. thaliana* DEN1 deNEDDylase regulates the pool of free NEDD8 and impacts on the AXR1/NCE2 NEDDylation machinery. An essential function of *C. elegans* NEDP1 deNEDDylase in DNA damage-induced apoptosis was presented by Dimitris Xirodimas (France) and discussed its role in poly-NEDD8 chains and the formation of the death promoting complex ‘apoptosome’ upon genotoxic stress. Similar to poly-NEDD8 chains, SUMO2/3 chains have been implicated in forming dynamic signaling platforms. Dimitris Liakopoulos (France) reviewed the multi-layered function of SUMOylation in yeast cell cycle and discussed how SUMO-targeted ubiquitin ligases are recruited to SUMO-modified kinetochores, ensuring correct anaphase-spindle positioning and elongation. The implication of SUMO-mediated protein interactions in plant development and stress responses was addressed by Beatriz Orosa (UK), showing a direct link between SUMOylation and plant growth control. Focusing in the same area, Maria Lois (Spain) presented a mechanism whereby SUMOylation regulates leaf senescence.

## Dissecting DUB mechanism and their regulation in model systems

DUBs play a critical regulatory role in counteracting ubiquitylation and understanding their regulation is paramount. The catalytic activities of USP1, USP46, and USP12 are modulated by distinct binding partners. Ning Zheng (USA) presented molecular studies of USP12, revealing that UAF1 and WDR20 potentiate USP12 catalytic activity. Further structural studies of DUBs were presented by Isabelle Jupin (France), unravelling a dual DUB/protease activity associated with the Turnip yellow mosaic virus (TYMV)-encoded precursor replication polyprotein (PRO).

Often functionally associated with the ZOMES, DUBs are actively studied in a number of systems, including immune signaling, regulation of endocytic processes, and regulation of receptor turnover. An intriguing example presented by Michael Naumann (Germany) is USP48, directly interacting with CSN in the nucleus, regulates the turnover of active NF-κB/RelA. DUB regulation by controlling the right subcellular ‘site of action’ was discussed by Erika Isono (Germany), investigating the metallo-DUB AMSH3 in ubiquitin-dependent endocytotic protein degradation in plants. Carlos Niño (Italy) presented another example of DUB interplay, where USP25 regulates the balance of EGFR recycling *versus* lysosomal degradation.

## ZOMES in ageing and disease

The pleiotropic functions of the ZOMES have unsurprisingly linked them to health processes. With recent advances in the understanding of the ZOMES through ground-breaking molecular studies, the field is maturing to translate this knowledge in health. CSN has an established role in promoting carcinogenesis and cancer progression. Mong-Hong Lee (USA) demonstrated that CSN6 plays a prominent pro-proliferative role in the EGFR-ERK signalling cascade. While CSN5 was recently shown to be a critical link between inflammation and cancer progression, Juergen Bernhagen (Germany) highlighted an atheroprotective role of CSN5 through modulating the NF-κB immune response pathway.

The implication of the ZOMES to health is not limited to the CSN and extends to other players of the UPS. Gerry Melino (UK, Italy) presented an overview on E3 ligases regulating the turnover of p53 tumour suppressor family members and the functional role of WWP1 ligase in acute myeloid leukaemia. Aging-related diseases are frequently associated with deregulated UPS. Shenhav Cohen (Israel) showed her work aiming at understanding myofibril breakdown in the context of UPS-dependent myofibril degradation in muscle atrophy. Simona Polo (Italy) recent work suggested a direct role of the *D. melanogaster* orthologue of HECW1 ubiquitin ligase in autophagy and RNA processing. Using the *C. elegans* ageing model, Niki Chondrogianni (Greece) presented the evaluation of proteasome-activating compounds able to extend the life-span of the worm. Bringing the research on the ZOMES to new heights with promises of eternal youth to ZOMEists and co-workers!

## Publisher’s Note

Springer Nature remains neutral with regard to jurisdictional claims in published maps and institutional affiliations.

